# Editorial: The role of microbiome in sustainable agriculture

**DOI:** 10.3389/fmicb.2024.1388926

**Published:** 2024-03-21

**Authors:** Songlei Xue, Ling Kui, Rouhallah Sharifi, Jian Chen

**Affiliations:** ^1^Jiangsu Coastal Area Institute of Agricultural Sciences, Yancheng, China; ^2^Guangxi Key Laboratory of Medicinal Resources Protection and Genetic Improvement, Guangxi Medicinal Botanical Garden, Nanning, China; ^3^Shenzhen Qianhai Shekou Free Trade Zone Hospital, Shenzhen, China; ^4^Department of Plant Protection, College of Agriculture and Natural Resources, Razi University, Kermanshah, Iran; ^5^International Genome Center, Jiangsu University, Zhenjiang, China

**Keywords:** microbiome, sustainable agriculture, plant, soil, stress

Modern agriculture is pressed by direct need for genetic improvement and sustainability to meet the food requirements of ever increasing global population. Additionally, the unpredicted climatic variations leading to droughts, heatwaves, and floods have negatively affected plant health and yield. Moreover, diseases, pests, and weeds also compromise plant performance. Taken together, these factors severely compromise health, performance, yield, and adaptability of crop species, which has serious consequences on global human population as well as livestock. Therefore, coordinated efforts are needed to ensure plant health, without compromising environment and climate. Both aerial and underground plant parts are associated with a myriad of microbial species, together called microbiome. These microbial species interact with plants and may provide beneficial functions to their hosts including tolerance to biotic and abiotic stress, nutrient use efficiency, water use efficiency, etc. The use of microbiomes in enhancing plant performance and health under adverse conditions has been the focus of research in modern agriculture. The microbiome research leading to sustainable agriculture can be categorized into different segments viz elucidation of molecular details of plant-microbe and microbe-microbe interactions, spatial and temporal profiling of microbiomes across diverse conditions and growth stages, developing culturing techniques for beneficial microbes, microbiome engineering to create synthetic communities with beneficial functions for host species under adverse conditions, and advancements in omics technologies to facilitate in-depth microbiome research. Modern research provides ample evidences to support the intelligent use of microbial species for enhanced plant performance as well as tolerance to biotic and abiotic stresses (Chen et al., 2022). Given the importance of microbiome in sustainable agriculture, it is essential to decode the complicated and interwoven ecophysiology of plant-microbe interaction. Upon exposure to biotic and/or abiotic stress, plants trigger certain physiological and molecular pathways to cope up with the prevailing conditions. The microbiome can potentially divert the plant metabolism toward these pathways to ensure better plant performance and health under adverse conditions (Chen et al., 2022). Additionally, the microbes may aid in, mineralization, solubilization, and mobilization of nutrients. Besides, they can produce siderophores, antibiotics, enhanced release of plant growth-promoting substances etc. The most attractive advantage of using microbiomes is their neutral impact on environment due to which the demand for agricultural chemical products such as pesticides, insecticides, and chemical fertilizers is expected to decline, while that of agricultural microbial products is anticipated to rise rapidly with time (Singh et al., 2020). Omics technologies have greatly facilitated the microbiome research, and we expect that with the advancement of sequencing technologies and bioinformatic pipelines, the identification and characterization of precise microbial species improving plant performance and health under adverse conditions would improve, which can lead to more strategic, novel and efficient strategies for sustainable agriculture. Amplicon sequencing facilitates the elucidation of microbiome composition as well as its organization and spatial distribution. This approach has successfully provided extensive insights about the microbiota residing in different niches in diverse crop species including rice, millet, soybean, corn, barley, wheat, sugarcane, pea, cucumber, grapevine, etc. Additionally, whole genome shotgun sequencing approach resulting into metagenome-assembled genomes (MAGs) and gapless circularized MAGs (cMAGs) allow researchers to gain deeper insights about the evolution and functional importance of microbiomes associated with plants (Trivedi et al., 2021). To gain proper understanding regarding the interactions between plant and microbes, information from other high-throughput omics technologies such as transcriptomics, proteomics and metabolomics shall be incorporated with the metagenomic data. Finally, there is need for proper culturing of microbial species to be commercially effective in sustainable agriculture. Once the cultural species are available, functional characterization and validation of genes involved in plant-microbe and microbe-microbe interaction, signaling pathways, colonization etc. can be uncovered (Trivedi et al., 2021). This editorial is based on a collection of eight articles focusing on structural and compositional profiling of microbiome across different growth stages and conditions, as well as characterization of beneficial functional role of microbial species.

The establishment and enrichment of beneficial microorganisms is influenced not only by the genotype of the plant and inter-species microbial interactions but the environmental conditions, the agricultural practices, and growth stages also play a substantial role in determining the final microbiome profile of a plant. Therefore, the spatial microbiome profile of each crop species under these factors needs to be developed to provide a solid platform for ensuring sustainable agriculture. Accordingly, one research article focused on the elucidation of the dynamics of endophytic microbial structure across the life cycle of the ratooning rice Jiafuzhan (Dong et al.). The growth stages chosen were seedling, tillering, jointing, heading, and mature stages of the first crop. Furthermore, the authors followed the experiment till the second crop and included the growth stages of 13, 25, and 60 days after regeneration at the heading, full heading, and mature stages, respectively. The 16S rRNA and ITS amplicon sequencing approach was followed to study the dynamics of microbial community structure. The authors observed that *Bradyrhizobium*, a nitrogen-fixing genus, occurred at all growth stages. Furthermore, the α-diversity and β-diversity of root endophytic bacteria and fungi vary considerably across different growth stages. The study identified biomarker bacteria for each growth stage. While the Proteobacteria, Actinobacteria, Bacteroidetes, Acidobacteria, and Spirochaetes were the most dominant bacterial phyla across all the stages, the most dominant fungal phyla included Ascomycota, Basidiomycota, Mortierellomycota, Rozellpmycota, and Mucoromycota. However, their abundance varied at different growth stages with Proteobacteria most abundant at tillering stage and Ascomycota most abundant at 13-day regeneration stage (Dong et al.). Furthermore, the seedling stage was dominated by *Pseudomonas, Hydrogenophaga, Flavobacterium*, and *Uniginosibacterium*; the tillering stage was dominated by *Halomonas* and *Cupriavidus*; the jointing stage was dominated by *Sulfuritalea, Candidatus, Koribacter, Roseimarinus*, and *Treponema*; Heading stage was dominated by *Actinobacteria* and *Streptomyces*, and the mature stage was dominated by *Geobacter* and *Bradyrhizobium*. Similarly, at the regeneration stage, *Dechloromonas, Anaerobacterium, Treponema, Desulfovibrio, Pleomorphomonas, Rhizomicrobium*, and *Dongia* were dominant at 13-day stage, *Leptonema and Ellin6067* were dominant at 25-day stage; and *Novosphingobium, Afipia, Acidibacter, Dokdonella, Sphingobium, Haliangium, Ancalomicrobium, Subgroup 10, Thiobacillus*, and *Devosia* were dominant at 60-day stage (Dong et al.). The findings of this study are important as it may facilitate further studies to uncover the most potent species with significant beneficial impact at each growth stage of the species. These microbial species can then be used as biofertilizer, specific for each growth stage to achieve the most enhanced results in terms of plant performance.

The ultimate goal of scientific findings is to translate them to provide a practical solution to human problems. One step ahead of structural profiling and functional characterization of plant microbiomes is to translate them into a useful product. Therefore, it is essential to evaluate and characterize the microbial species in terms of their beneficial aspects on the host species. Once, the beneficial functional role of microbes is established, the species can be harnessed to translate them into a technological product. Accordingly, another research article focused on the elucidation of growth promoting potential of an endophytic *Rhizobium* sp. WYJ-E13 strain from the roots of a medicinal plant, *Curcuma wenyujin* (Huang et al.). The authors studied the morphological characteristics along with molecular characterization using 16S rDNA sequencing. Following inoculation, they confirmed the colonization of the bacterium in the root through scanning electron microscopy and found that inoculated samples exhibited a significant increase in growth rate, seedling height, and root length. Furthermore, the growth promoting effect could be due to production of cytokinin by the bacterium. To further decode the functional relevance of the bacterium, the authors developed deeper genomic insights through whole genome sequencing, which revealed a 4.35 Mb circular chromosome and two plasmids. A total of 4,349 protein coding genes were identified, many of which were found to be involved in growth promoting function including the genes involved in nitrogen metabolism, hormone production, sulfur and phosphate metabolism, and root colonization (Huang et al.). Taken together, the insights generated from this study are useful to develop a microbiome based biofertilizers from WYJ-E13 strain, however, further research is required to evaluate its efficacy under different conditions. Furthermore, a different study focused on the potential of a fungal endophyte *Epichloë bromicola* from wild barley to confer salt tolerance in cultivated barley (Wang et al.). Following inoculation of barley seeds, the growth of endophyte colonies was traced and monitored microscopically. The endophytes showed successful inheritance to next generation seeds, which were then studied for morphological characteristics and molecular characterization. The authors found that the inoculated plants exhibited enhanced plant height, shoot biomass, and total biomass under salt stress. Furthermore, increase in chlorophyll content and osmolyte synthesis was observed. Moreover, the authors also found significant differences in metabolite accumulation between inoculated and non-inoculated barley plants under salt stress (100 mM and 300 mM), which may confer stress adaptation to host, especially those involved in glutathione and nitrogen metabolism, flavone and flavonol biosynthesis, ABC transporters, lysine degradation, arginine and proline metabolism, and amino acids biosynthesis (Wang et al.). This study provided insights that salt hardier cultivated barley germplasm could be produced by transferring endophytic fungi from wild species.

In addition to the factors mentioned above that may cause variations in microbial communities associated with host species, anthropogenic activities such as application of fungicides, insecticides, and fertilizers could also lead to significant changes in community structure (Chen et al., 2022). In this regard, one article focused on the impact of nitrogen, phosphorus and potassium (NPK) fertilizer application on structural dynamics of root microbiota community of oil palm seedlings (Ding et al.). The authors found the enhanced growth of the Pseudomonadota and Bacteroidota due to fertilizer application, which prefer nutrient-rich conditions for growth. However, the microbiota profile significantly depleted when the soil was sterilized. The seedlings grown in unsterilized soil and with fertilizers showed enhanced growth parameters than those grown in unfertilized soil. Despite the fertilizer application, the seedlings grown in sterilized soils showed stunted growth that may be due to excessive accumulation of soluble salts in the root zone and reduced the number of native soil microbes. Sterilization reduced the soil microbial species, however, they re-established themselves latter but the microbial diversity was higher in fertilized normal soil compared with fertilized sterilized soil. Additionally, re-established microbial communities were distinct from the original indigenous microbiota. The authors suggest the re-establishment of the microbial communities in pre-sterilized soils may be due to irrigation, rain, human activity or insect activity. Taken together, these results suggest that fertilizer application to normal soils may divert indigenous soil microbial profile toward improved nutrient acquisition or transformation for enhanced plant performance (Ding et al.). More research is required to functionally characterize the exact role of these native soil microbes and to uncover their impact on nutrient acquisition or transformation. Soil sterilization and fertilizer application may have a negative effect on the growth and microbiome composition of the oil palm seedlings, as depletion of several microbes belonging to different categories was observed, which could significantly affect plant fitness and soil microbiome stability (Ding et al.). Additionally, land use, tillage, crop rotation, and irrigation, and agricultural practices also alter the microbiome profile (Chen et al., 2022). A different study tried to uncover the microbiome profile of soybean rhizosphere under nature farming i.e., cultivating crops without using chemical fertilizers and pesticides (Agyekum et al.). The authors tested the hypothesis that nature farming enables a distinct and diverse rhizosphere microbiome to enhance plant performance. Instead of using chemical fertilizers and pesticides, nature farming entails crop rotation and green manuring to maintain soil fertility and crop growth. In contrast to organic farming, no untreated animal manure and urban sewage are employed in nature farming, as they may be potential source of pathogenic contamination. The study revealed that nature farming not only encompassed higher microbial diversity but also exhibited abundance of beneficial soil microbes. The wild-type and non-nodulating mutant soybean plants were grown in conventional and nature farming soils. Furthermore, both soil types were treated with fumigant to analyse its effect on microbial communities and growth of soybean plants. The shoot lengths of wild type plants were significantly higher compared to mutant type under both farming systems and fumigation. In contrast to the conventional farming, application of chloropicrin fumigant under nature farming significantly decreased the number of both seed pods and seeds pods in wild-type soybean. The reason for such effect could be due to the greater impact of fumigation on nitrogen-fixing rhizobia. Besides, the non-nodulating mutant soybean plants showed increased seed number upon fumigation under conventional farming, while an opposite effect was observed under nature farming conditions. Therefore, soil fumigation poses significant influence on diversity of soybean rhizosphere microbiome under different farming systems, with *Rhizobium, Mesorhizobium, Streptomyces*, and *Burkholderia* enriched with non-fumigated and *Herbaspirillum, Amycolatopsis*, and *Bosea* enriched in fumigated soils. Furthermore, both farming system also influences bacterial and fungal communities, with Firmicutes most abundant phyla in the conventional farming and those of Acidobacteria and Bacteroidetes dominant in the nature farming system. While the *Agrobacterium, Bacillus*, and *Cupriavidus* were the abundant genera in the conventional farming, *Rhizobium* was dominant in nature farming system. Likewise, *Mortierella* and *Trichoderma* were abundant fungal genera in the conventional farming, while *Clonostachys* and *Metarhizium* dominant under nature farming. Moreover, there were significant differences in abundance of bacterial and fungal taxa under farming system and soil fumigation. *Rhizobium* and *Phenylobacterium* were particularly abundant under nature farming rhizosphere soil, while *Bacillus sp*., *Cupriavidus*, and *Paenibacillus* were enriched in conventional farming soil (Agyekum et al.).

Elevation gradient represents an excellent ecological parameter to study the variation and relationship of host associated microbiomes with changing environments. The microbial composition associated with plants, besides other factors, is also influenced by ecological factors including altitude. *Dendrobium nobile*, a Chinese medicinal plant, was metabolically characterized and differences in metabolite abundance, particularly fatty acids were observed across different altitudes (Zhao et al.). Furthermore, evaluation of microbial species associated with *D. nobile* across different altitudes revealed significant differences. Based on their results, the authors suggested that metabolism and development of *D. nobile* across different altitudes was affected through induction of fatty acid metabolites and plant hormones biosynthesis (Zhao et al.). With increased elevation, the Ascomycote endophytic fungi were abundant, while those of Basidiomycota tended to decrease. The study concluded *Toxicocladosporium rubrigenum* as the key endophytic fungus linked to higher altitudes. Besides, fatty acid composition also increased with increasing altitude. Some of the endophytic fungi associated with *D. nobile* at high altitude were found to be correlated with upregulation of fatty acid metabolites. The authors observed that the higher altitude manifests biomarker endophytic fungi. Besides, higher altitude significantly leads to enhanced synthesis and accumulation of fatty acids including those involved in JA synthesis pathway (Zhao et al.).

Finally, among the two review articles, one focuses on the microbiome changes across different life cycle stages of sugar beet (from seed to beet) as well as the approach for microbiome management (Wolfgang et al.). The review provided a foundation and baseline to gain deeper insights about sugar beet microbiome research and to facilitate further research on how sugar beet rhizosphere can be modulated for biocontrol of pathogens. The other review focused on the nature of the relationship between common mycorrhizal networks (CMNs) assisted by arbuscular mycorrhizal fungi (AMF) and plant species (Ullah et al., 2024). Regardless of the plant species, the AMFs colonize and form extensive networks with numerous neighboring plants for their carbon or nutritional supplies. Accordingly, the resources are distributed either more evenly across all the connected plants or the resources are directed particularly toward a specific host. The authors have drawn an excellent similitude to these two scenarios, with former termed as socialistic approach and the latter as capitalistic approach. Socialistic association is established during phytohormone mediated growth stimulation, mitigating biotic and abiotic stresses, as well as direct or indirect transfer of nutrients to plants. The capitalistic approach is ensured when there is competition between different plants and the victor species tame the AMFs for their own advantage. The authors have portrayed the ubiquity, functional dynamics of plant-plant and plant-microbe interactions in CMNs, and role in promoting plant performance under adverse environmental conditions as socialistic or capitalistic picture (Ullah et al., 2023).

## Author contributions

SX: Writing – original draft. LK: Writing – original draft. RS: Writing – review & editing. JC: Writing – review & editing.
